# Late onset of biliopleural fistula following percutaneous transhepatic biliary drainage: a case report

**DOI:** 10.1051/bmdcn/2018080106

**Published:** 2018-02-26

**Authors:** Edward Yi-Yung Yu, Fei-Shih Yang, Yu-Jen Chiu, Fuu-Jen Tsai, Chi-Cheng Lu, Jai-Sing Yang

**Affiliations:** 1 Department of Radiology, MacKay Memorial Hospital Taipei 104 Taiwan; 2 Department of Radiology, Taitung MacKay Memorial Hospital Taitung 950 Taiwan; 3 Division of Reconstructive and Plastic Surgery, Department of Surgery, Taipei Veterans General Hospital Taipei 112 Taiwan; 4 Genetics Center, Department of Medical Research, China Medical University Hospital Taichung 404 Taiwan; 5 School of Chinese Medicine, China Medical University Taichung 404 Taiwan; 6 Department of Medical Genetics, China Medical University Hospital Taichung 404 Taiwan; 7 Department of Pharmacy, Buddhist Tzu Chi General Hospital Hualien 970 Taiwan; 8 Department of Medical Research, China Medical University Hospital, China Medical University Taichung 404 Taiwan

**Keywords:** Biliopleural fistula (BF), Percutaneous transhepatic biliary drainage (PTBD), Neuroendocrine tumor, Jaundice

## Abstract

Biliopleural fistula (BF) and formation of biliopleural effusion is a rare complication following percutaneous transhepatic biliary drainage (PTBD). It occurs when the pleura is traversed by the catheter before entering the bile duct. Biliopleural fistula should be suspected when right side pleural effusion develops following the PTBD procedure. The diagnosis of biliopleural fistula is made when greenish pleural fluid with high concentration of bilirubin is aspirated. Here we present a case where a patient develops a biliopleural fistula following PTBD due to obstructive jaundice caused by neuroendocrine tumor of pancreas. Biliopleural fistula was disclosed after a scheduled catheter replacement procedure. Treatments of biliopleural fistula include thoracentesis with drainage tube installation into pleural space. In addition, a drainage tube was installed through percutaneous transhepatic gallbladder drainage (PTGBD) to reduce the bile induced pressure. Surgical repair of fistula was performed after the conservative treatment was unsuccessful. The patient expired 5 days after surgery due to respiratory failure.

## 1. Introduction

Biliopleural fistula (BF) and the formation of biliopleural effusion is a rare complication of percutaneous transhepatic biliary drainage (PTBD) [1-3]. It occurs when the pleural cavity is traversed during the procedure to gain access to the biliary tract. The likelihood of fistula formation increases with the duration of catheter placement [4].

Here we present a case of biliopleural fistula which was disclosed 98 days after the initial PTBD procedure. Our objective of this case is to increase the awareness of interventional radiologists of this rare complication that may lead to significant morbidity and even mortality.

## 2. Case Presentation

A 53-year-old woman with a clinical history of neuroendocrine tumors in pancreas head with hepatic metastasis was admitted to our center due to recent onset of jaundice. Abdominal computed tomography (CT) revealed pancreas head tumor, multiple hepatic metastasis and dilatation of intra hepatic bile duct (IHD) and common bile duct (CBD).

**Fig. 1:**
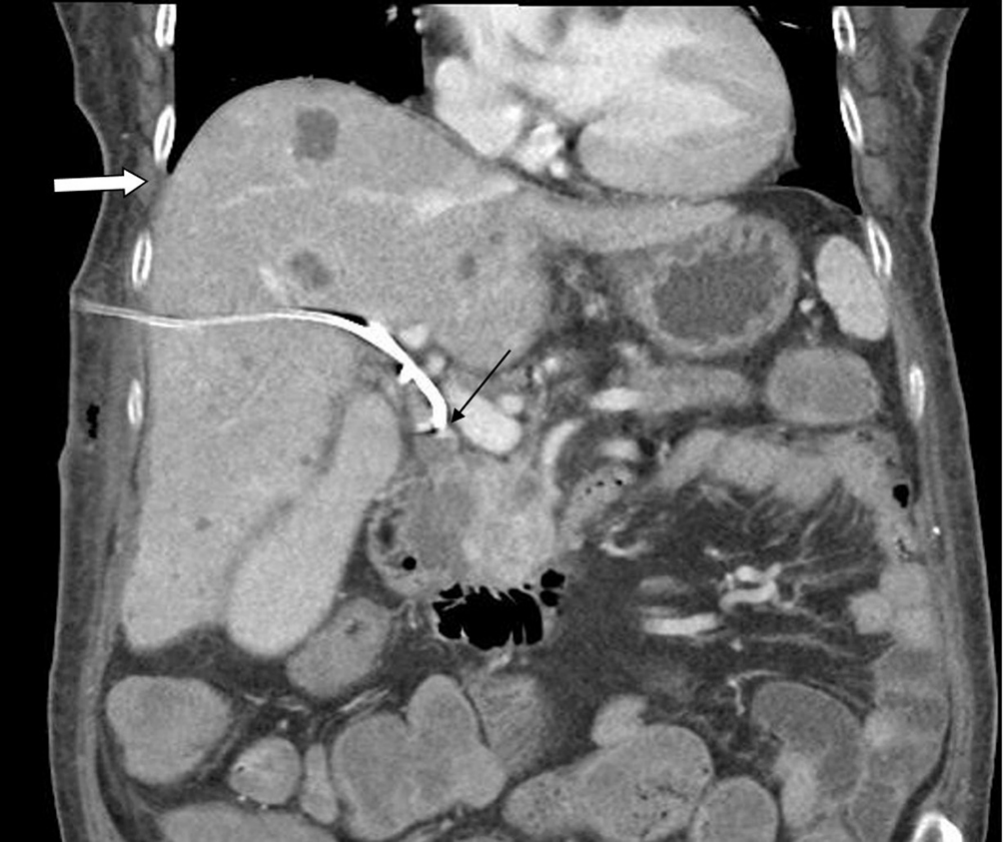
Coronal view of the post enhancing abdominal computed tomography reveals the tip of the catheter in the common bile duct (thin arrow), and without right pleural effusion (thick arrow).

**Fig. 2:**
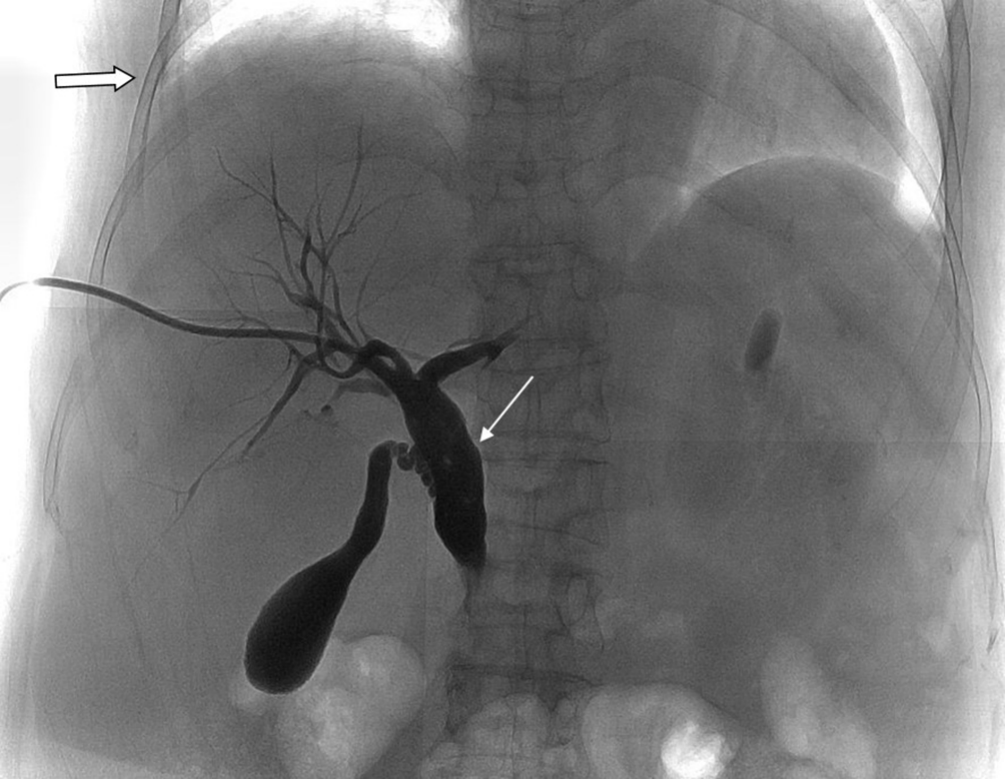
Cholangiogram reveals the tip of the catheter in the common bile duct (thin arrow). The right costophrenic angle is well demonstrated (thick arrow).

**Fig. 3:**
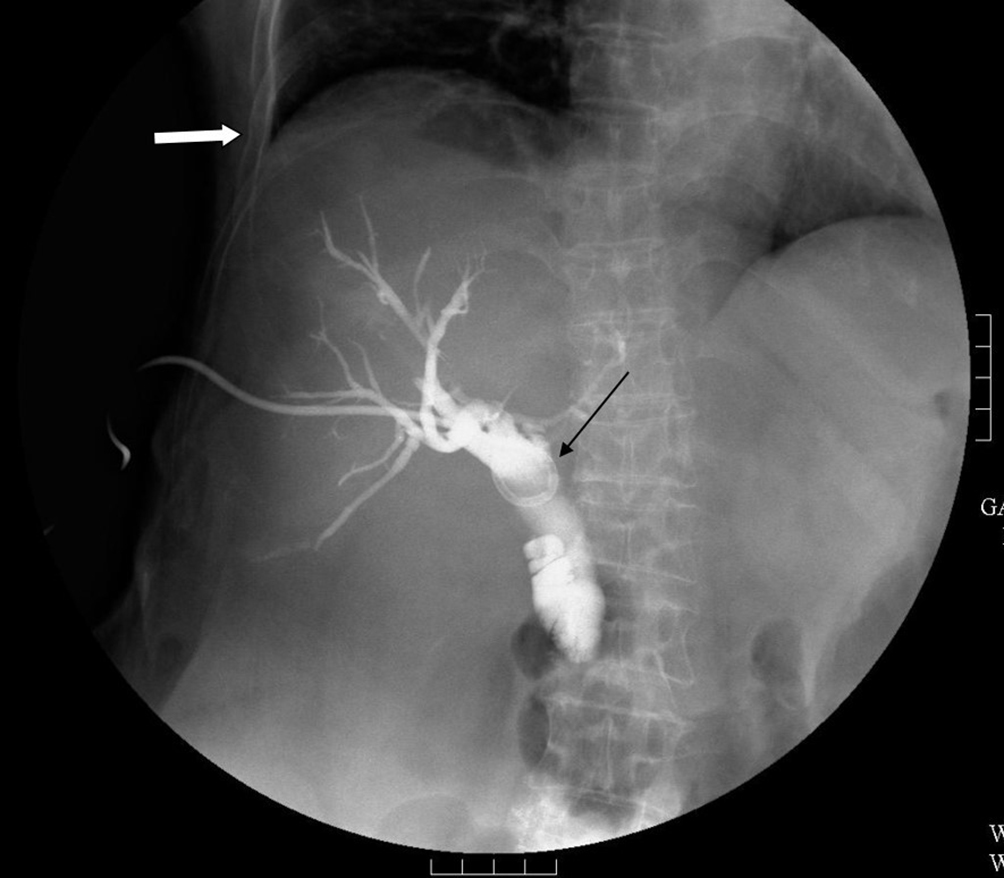
Post catheter replacement cholangiogram reveals the tip of the catheter in the common bile duct (thin arrow). The right costophrenic angle is well demonstrated (thick arrow).

Percutaneous transhepatic biliary drainage (PTBD) was performed as a palliative treatment to reduce bile induced jaundice. Right side transhepatic approach was performed with a needle inserted through midaxillary line between 9th and 10th rib. An 8 French (FR) multiple side hole, a pig-tailed was inserted into CBD for continuous drainage. Abdominal CT was arranged the next day, and there was no evidence of procedure-related complications (Fig. 1). The positioning of the catheter was followed up regularly with chest X-ray film and plain abdominal film without evidence of catheter migration. A follow up PTBD was performed 45 days after the initial PTBD, and without evidence of catheter migration (Fig. 2). Scheduled catheter replacement was arranged 98 days after the initial PTBD procedure. A new catheter the same caliber was inserted into the CBD without technical difficulty (Fig. 3).

**Fig. 4:**
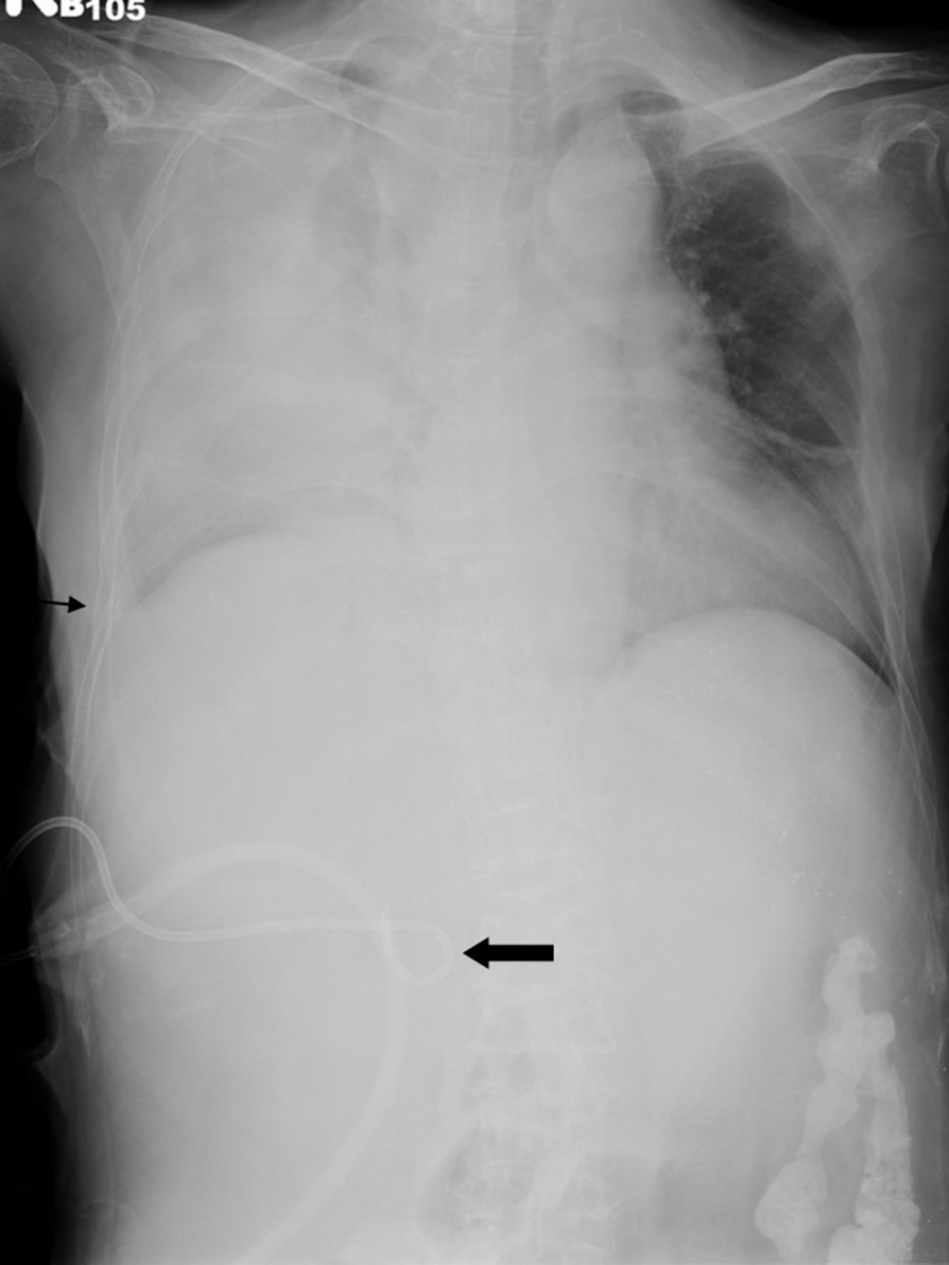
Chest X-ray film reveals opacification of the right lung, blunting of the right costophrenic angle (thin arrow) and partial migration of the catheter (thick arrow).

This patient developed high fever 8 days after returning to the ward. Chest X-ray film revealed right side pleural effusion (Fig. 4). Thoracentesis was performed and greenish fluid was collected. A drainage tube was installed into right pleural space for continuous drainage. Biochemical report of the greenish fluid revealed a high concentration bilirubin (23.7 mg/*dl*). Bacteria culture revealed *Enterococcus* and *Candida albicans*. Appropriate antibiotics were given accordingly.

**Fig. 5:**
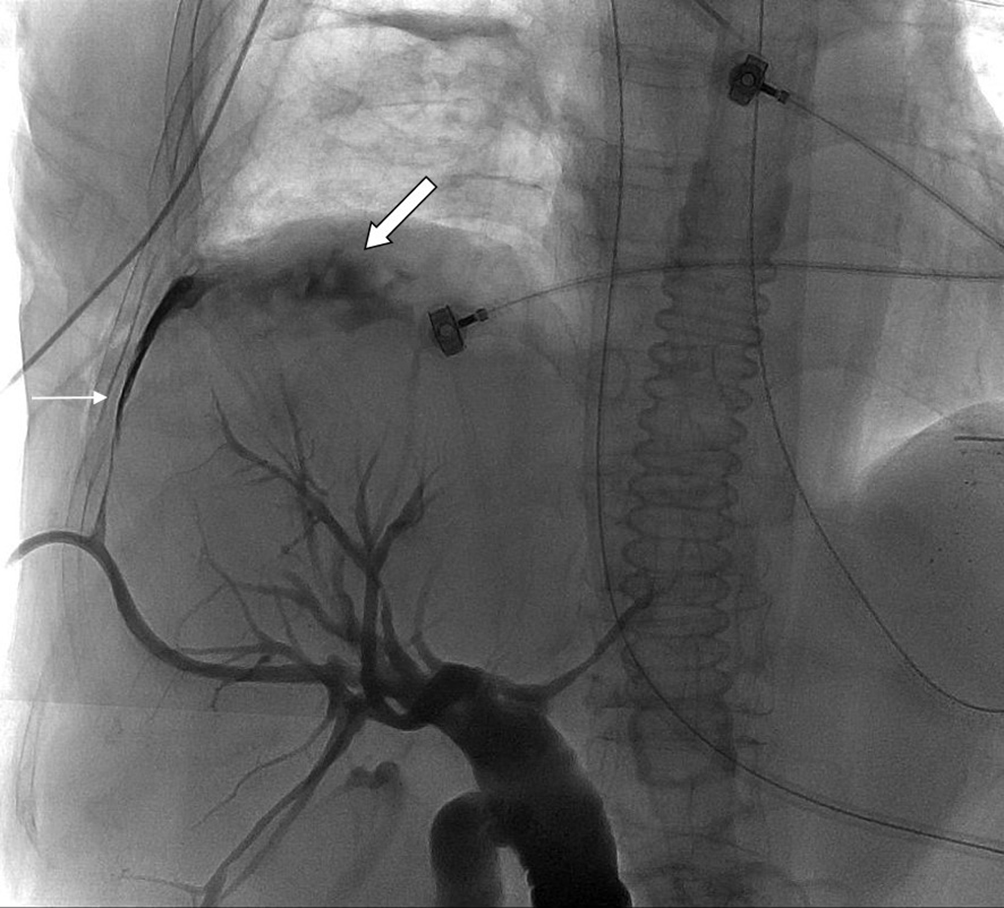
Cholangiogram reveals biliopleural fistula (thin arrow) with back flow of contrast media into the right pleural space (thick arrow).

Follow-up of PTBD was performed the next day, and BF with back flow of contrast media (CM) into right pleural space was disclosed (Fig. 5). Additional installation of drainage tube *via* percutaneous transhepatic gallbladder drainage (PTGBD) was performed 3 days later due to no sign of decreasing drainage amount of right pleural effusion. Surgical repair of the fistula was arranged 4 days later due to persistent right pleural effusion. The patient expired 5 days after surgery due to respiratory failure.

## 3. Discussion

Percutaneous transhepatic biliary drainage is often used in treatment of obstructive biliary disease to relieve symptoms prior to surgery or palliatively in patients who are poor candidates for surgery [1]. Biliopleural fistula is a rare complication following this procedure, which may be due to the passage of catheter through the pleural cavity before crossing the diaphragm and into the bile duct when a transhepatic approach is used [1, 5-7]. Elevated pressure gradient in the biliary tract could drive the bile leak back into the pleural cavity. The likelihood of fistula formation between the biliary tract and pleural cavity increases with the duration of catheter in place and is the primary factor leading to fistula formation [4]. Studies have shown that fistula formation occurs within 3 weeks of catheter placement and biliopleural effusion may develop when the catheter remained in bile duct for more than 4 weeks [1]. One study revealed common features that lead to the development of the BF: (i) Complete biliary obstruction was present; (ii) Catheter placement was between the 9th and 10th ribs in the midaxillary line; (iii) prolonged drainage (7 days to 2 months) preceded fistula formation [7]. These features are also seen in our patient. Another study suspected the path created by the large drainage tubes served as an ideal passage through which bile could leak back into the pleural cavity in the presence of persistent biliary tract obstruction [7]. Bilious fluid collections can be present anywhere along the path of the PTBD catheter from the biliary tree to the pleural space [7].

We suspect the cause for the late onset of BF and biliopleural effusion in this patient: (A) high efficiency of the catheter in draining the bile juice from CBD; (B) the residual blood clot and the infected bile juice with much debris accumulated at the path of the catheter from the initial PTBD procedure, which acts as a sealant forming between the catheter and the adjacent liver tissue to prevent the back flow of bile juice from the drainage tract into the pleural space. Removal of the original catheter causes the sealant to dislodge from the drainage tract; this creates a tiny space between the new catheter and the adjacent liver tissue, leading to the back flow of bile juice from CBD, around the catheter, and into pleural space.

A high percentage of patients in other series and in our patient developed empyema. The reasons for infectious complications are multiple. The direct tract from the skin to the pleura without true pleural drainage could predispose to pleural seeding with bacterial pathogens [1]. The incidence of cholangitis in patients with completely obstructed and dilated biliary tracts is high (> 80%) without clinical symptoms [7]. The patient should be evaluated for empyema if a BF develops.

Early diagnosis of BF can reduce complications requiring surgery [1]. The diagnosis of BF should be suspected when a patient with a PTBD catheter develops a right pleural effusion. Ultrasound, CT and radionuclide scan can identify bile collection, but they cannot determine fistula location [7]. Confirmation is obtained on thoracentesis when bilious green fluid aspirated that has a pleural fluid total bilirubin to serum total bilirubin ratio >1.0 [4]. Follow-up cholangiogram may sometimes reveal the fistulous tract when the CM regurgitates through the side hole of the catheter and into the pleural cavity.

The treatment for BF is surgical; although few patients tolerate surgical intervention, it can also be treated with conservative measures [1, 7]. Bile drainage from the pleural cavity can be done conservatively with a drainage tube. Early institution of another form of biliary drainage appears to be the single most important factor in the successful management of BF [4]. One study with surgical experience with traumatic BF revealed that chest drainage ceases or dramatically decreases once an alternative route of biliary decompression is available [7]. Surgery is required if the fistula is large or diseased tissue must be debrided [1]. The application of antibiotics as an adjunct to bile drainage is critical because: (i) bacteria can enter along the catheter path; (ii) bile is often infected; (iii) bile stasis encourages bacteria growth [4]. Because the likelihood of fistula formation is related to the length of time the catheter is in place, prevention relies on decreasing the duration of the time the catheter is in place.

## 4. Conclusion

Biliopleural fistula and biliopleural effusion is a rare but serious complication of PTBD. It should be suspected in patient who develops right side pleural effusion after PTBD. This complication should be treated conservatively, and surgical treatment should be performed if the conservative treatment has failed.

## Declaration of Conflicting Interests

The author(s) declared no potential conflicts of interest with respect to the research, authorship, and/or publication of this article.
